# Molecular approach to food allergy diagnosis and therapy

**DOI:** 10.1186/2045-7022-1-S1-S28

**Published:** 2011-08-12

**Authors:** Ronald van Ree

**Affiliations:** 1 Academic Medical Center at the University of Amsterdam, Amsterdam, Netherlands

## 

Over the past decades, major allergens of the most important allergenic foods have been identified, isolated, cloned and expressed as recombinant molecules. This development has sparked off a revolution in allergology towards molecular approaches for diagnosis and therapy. The roles of individual molecules in clinical phenotypes are being elucidated rapidly. One of the first clear examples that individual molecules are associated with clinical phenotypes is allergy to Rosaceae fruits such as apple and peach. We now know that fruit allergy caused by pollen-related PR-10 proteins and profilins is mild and restricted to the oral cavity. On the other hand, fruit allergy caused by lipid transfer proteins is often at the basis of more severe systemic reactions. Molecular diagnostics based on such observations are a promis for the future and will increasingly replace extract-based diagnostics. Availability of recombinant major food allergens has also revived the interest in immunotherapy for food allergy. First attempts towards the end of the last century to treat food allergy with aqueous extracts were upset by extremely high incidence of side-effects. The advent of recombinant technology now allows evaluation of safer approaches of immunotherapy for food allergy. Recombinant food allergens can be modified into hypo-allergenic molecules that do not cause unacceptable levels of side effects. In 2008, the European Commission decided to fund a consortium to develop hypo-allergenic molecule-based immunotherapy for the treatment of fish and fruit allergy. The FAST project has now selected the most promising hypo-allergenic variants of fish parvalbumin and peach lipid transfer protein. GMP production has started and toxicity studies and Phase I trials are being planned. The coming years will learn whether these approaches meet the expectations to provide a safe and effective treatment for food allergy.

